# Therapeutic Potential of Venetoclax and Selinexor in Targeting Hypoxia‐Induced Vulnerabilities in Multiple Myeloma

**DOI:** 10.1002/cnr2.70249

**Published:** 2025-06-20

**Authors:** Seiichi Okabe, Yuya Arai, Yuko Tanaka, Akihiko Gotoh

**Affiliations:** ^1^ Department of Hematology Tokyo Medical University Tokyo Japan

**Keywords:** BCL2, hypoxia, myeloma, selinexor, venetoclax

## Abstract

**Background:**

Multiple myeloma (MM) is a blood cancer marked by the abnormal clonal growth of plasma cells. Hypoxia plays a critical role in the progression and treatment resistance of MM.

**Aims:**

This study investigates the expression of B‐cell/CLL lymphoma 2 (BCL2) family genes. We also investigated the activity of BCL2 and exportin‐1 (XPO1) inhibitors and the potential therapeutic synergy of venetoclax and selinexor under hypoxic conditions.

**Methods and Results:**

Analysis of publicly available datasets revealed hypoxia‐induced upregulation of *BCL2* and *BCL2‐like 11* (*BCL2L11)*, while *BCL2‐associated agonist of cell death (BAD)* expression was suppressed. Venetoclax, a selective BCL2 inhibitor, demonstrated enhanced cytotoxicity and increased caspase‐3/7 activity under hypoxic conditions. Selinexor exhibited potent anti‐myeloma effects, including dose‐dependent reductions in cell viability and increased apoptotic activity. Combining selinexor with venetoclax under hypoxia produced anti‐myeloma effects, significantly reducing cell viability, increasing apoptosis, and disrupting the mitochondrial membrane potential. This combination effectively overcame resistance in bortezomib‐resistant MM cells and demonstrated efficacy in primary plasma cell leukemia (PCL) samples.

**Conclusion:**

These findings highlight the potential of selinexor and venetoclax combination therapy to exploit hypoxia‐induced vulnerabilities in MM cells.

**Abbreviations**
ANOVA: analysis of variance
BAD: BCL2 associated agonist of cell death
BCL2: B‐cell/CLL lymphoma 2
BCL2L11: BCL2 like 11
BIM: Bcl‐2 interacting mediator of cell death
BM: bone marrow
BTZ: bortezomib
FBS: fetal bovine serum
GEO: Gene Expression Omnibus
LDH: lactate dehydrogenase
MGUS: monoclonal gammopathy of undetermined significance
MM: multiple myeloma
MMP: Mitochondrial membrane potential
mRNA: messenger RNA
PBMC: peripheral blood mononuclear cell
PCL: plasma cell leukemia
PI: Propidium Iodide
RPMI 1640: Roswell Park Memorial Institute 1640
RT‐PCR: real‐time reverse transcription‐polymerase chain reaction analysis
SDs: standard deviations
XPO1: exportin‐1

## 1 Introduction

Multiple myeloma (MM) is a blood cancer marked by the abnormal clonal growth of plasma cells, which can lead to focal bone damage, kidney dysfunction, anemia, and elevated calcium levels [1]. Nearly all MM patients progress from an asymptomatic pre‐cancerous condition known as monoclonal gammopathy of undetermined significance (MGUS) [2]. Therefore, MGUS, a benign condition involving abnormal plasma cells, is a precursor to MM [3]. Although MGUS is typically regarded as benign, individuals with this condition face a heightened risk of developing MM or other plasma cell disorders, with a progression rate of about 1% annually [3]. In contrast, plasma cell leukemia (PCL) is a rare and highly aggressive form of MM that carries a poor prognosis [4]. PCL is marked by an unusually high number of plasma cells in the peripheral blood and differs from standard MM in its clinical presentation, cytogenetic characteristics, and resistance to treatment [4]. The processes driving the progression from MGUS to MM and eventually to PCL are poorly understood, but are believed to involve the stepwise accumulation of genetic mutations [5].

The treatment landscape for MM has transformed considerably over the past two decades, primarily due to the development of innovative therapies such as proteasome inhibitors and immunomodulatory drugs [6]. These developments have significantly enhanced patient outcomes, leading to prolonged overall survival and improved quality of life [6]. Exportin‐1 (XPO1) is widely recognized for its function in nuclear export. Recently, selinexor (XPOVIO, developed by Karyopharm Therapeutics) in combination with dexamethasone received approval for use in adult patients with relapsed or refractory MM [7]. Selinexor is a first‐in‐class oral selective XPO1 inhibitor that promotes apoptosis in cancer cells by causing the nuclear retention of oncogene messenger RNAs (mRNAs) and reactivating tumor suppressor proteins [8]. These advancements have resulted in higher overall survival rates and improved quality of life for patients. Despite this progress, MM remains an incurable cancer, with most patients eventually relapsing and needing additional lines of treatment [9]. Therefore, an alternative strategy is needed to improve the survival of myeloma patients.

MM cells are located in the bone marrow (BM), which features a highly structured architecture and contains various types of stromal cells [10]. The BM is a naturally hypoxic environment, with oxygen levels significantly lower than those found in other tissues, making hypoxia a defining feature of this niche [10]. As a result, various biological oxygen sensors and their signaling pathways are crucial in regulating hematopoietic processes in the BM under both normal and pathological conditions [11]. Therefore, the relatively low levels of oxygen in the BM may support MM progression. Moreover, the hypoxic niche offers a protective sanctuary for MM cells, safeguarding them from chemotherapy and facilitating the emergence of drug resistance mechanisms. Despite ongoing research into novel therapies and treatment strategies, including immunotherapies and targeted approaches, achieving a cure for MM is elusive.

Previously, the oral B‐cell/CLL lymphoma 2 (BCL2) inhibitor venetoclax has demonstrated promising efficacy in patients with t(11;14) plasma cell disorders, both as a single agent and in combination [12]. Thus, recent studies have explored the potential of targeting apoptotic pathways in MM, particularly through BCL2 family inhibition. The combination of BCL2 inhibitors with other targeted agents, such as XPO1 inhibitors, has gained interest as a potential therapeutic approach to overcoming resistance and enhancing treatment efficacy. As hypoxia influences myeloma cell survival and drug resistance, this study aimed to evaluate the efficacy of this combination therapy in promoting cell death under hypoxic conditions.

In this study, we investigated the proliferation of myeloma cells under hypoxic conditions, focusing on the expression of the *BCL2* gene, the BCL2 family member, BCL2‐like protein 11 (BCL‐2 interacting mediator of cell death: BIM), and BCL2 associated agonist of cell death (BAD). We examined the role of hypoxia in modulating these genes' expression and explored the therapeutic potential of combining venetoclax with selinexor.

## 2 Methods

### 2.1 Reagents

Selinexor (KPT‐330, ATG‐010), an XPO1 inhibitor, and venetoclax (ABT‐199, GDC‐0199), a BCL2 inhibitor, were obtained from Selleck Chemicals (Houston, TX, USA). All compounds were dissolved in dimethyl sulfoxide. Unless stated otherwise, all other reagents used in the experiments were sourced from Merck KGaA (Darmstadt, Germany).

### 2.2 Cell Lines and Cell Culture

The MM cell lines U266 (complex karyotype and aneuploid, with multiple chromosomal abnormalities, including translocations and deletions) and RPMI 8226 (complex karyotype and highly aneuploid, with multiple structural and numerical chromosomal abnormalities) were obtained from the American Type Culture Collection (ATCC, Manassas, VA, USA). The bortezomib (BTZ)‐resistant myeloma cell line KMS‐11/BTZ (t(4;14)(p16.3;q32.3) translocation) was also sourced from the Japanese Collection of Research Bioresources Cell Bank (Ibaraki, Osaka, Japan). All MM cell lines were maintained under standard culture conditions at 37°C in a humidified atmosphere using Roswell Park Memorial Institute 1640 (RPMI 1640) medium, supplemented with 2 mmol/L glutamine and 10% fetal bovine serum (FBS). MM cells were exposed to hypoxic conditions for specific experiments, maintaining oxygen levels at 1% O_2_ to simulate the hypoxic tumor microenvironment. In this study, normoxia was defined as 21% O_2_, reflecting atmospheric oxygen levels in a standard cell culture incubator, while hypoxia was set at 1% O_2_ using a hypoxia chamber with controlled gas regulation (1% O_2_, 5% CO_2_, 94% N_2_).

### 2.3 Primary PCL Sample

A primary PCL sample (complex karyotype: 41–43, add (2)(p14), add (3)(p24), add (7)(p21), −8, add (10)(p14), −13, −15, +1 ~ 2 mar) was also prepared for further study. With informed written consent, peripheral blood mononuclear cells (PBMCs) were isolated from the blood samples of patients with PCL using Lymphocyte Separation Medium 1077 (PromoCell GmbH, Heidelberg, Germany). The isolated PBMCs were cultured in a medium containing 20% FBS at a 5 × 10^5^ cells/mL density. Ethical approval for the study was granted by the Ethics Committee of Tokyo Medical University (approval number: T2023‐0105).

### 2.4 Data Collection and Processing

Microarray data were retrieved for analysis, specifically focusing on dataset GSE13591. GSE13591 encompasses gene expression profiles of purified plasma cells obtained from five regular donors, 11 MGUS patients, 133 MM patients, and 9 PCL patients at diagnosis. Additionally, we analyzed data from GSE80140. The GSE80140 study includes whole‐genome gene expression screening data for myeloma cell lines (RPMI8226, KMS‐11, KMS‐12BM, and MM.1S) subjected to chronic hypoxic conditions. Data were obtained from the Gene Expression Omnibus (GEO) database (https://www.ncbi.nlm.nih.gov/geo/) [13, 14]. Data analysis was performed using the GEO2R web tool, which enables interactive comparisons between various sample groups within GEO datasets. The data were downloaded in SOFT format, converted to XLS files, and processed using Microsoft Office Excel (Microsoft Corporation, Redmond, WA, USA). Differentially expressed genes were identified using an adjusted *p*‐value cutoff of < 0.05 and a log_2_ fold change threshold of ≥ 1.0 or ≤ −1.0.

### 2.5 Cell Viability Assay

MM cells were cultured at a density of 2 × 10^5^ cells/mL and treated with selinexor and/or venetoclax for 72 h. After treatment, cell viability was assessed using the Cell Counting Kit‐8 (Dojindo Laboratories, Mashikimachi, Kumamoto, Japan) following the manufacturer's instructions. Absorbance was measured at 450 nm using an EnSpire Multimode Plate Reader (PerkinElmer, Waltham, MA, USA).

### 2.6 Caspase 3/7 Activity

Caspase 3/7 activity was assessed using the Caspase‐Glo 3/7 assay kit (Promega Corporation, Madison, WI, USA), following the manufacturer's guidelines. MM cells were treated with specified concentrations of selinexor and/or venetoclax for 48 h. Luminescence was then measured using an EnSpire Multimode Plate Reader (PerkinElmer, Waltham, MA, USA) to quantify caspase activity.

### 2.7 Cytotoxicity Assay

Cytotoxicity was evaluated in MM cells treated with specified concentrations of selinexor and/or venetoclax for 48 h. The Cytotoxicity Lactate Dehydrogenase (LDH) Assay Kit (Dojindo Laboratories) measured the activity of LDH, an enzyme released during cell membrane damage that indicates cell death. A plate reader recorded the LDH released from dead cells, corresponding to the absorbance at 490 nm.

### 2.8 Quantitative Real‐Time Reverse Transcription‐Polymerase Chain Reaction Analysis (RT‐PCR)

Total RNA was isolated from MM cells using the RNAqueous‐4PCR Kit (Life Technologies Japan KK, Minato‐ku, Tokyo, Japan). Reverse transcription was carried out with the First‐Strand cDNA Synthesis Kit (OriGene Technologies, Rockville, MD, USA). Quantitative real‐time reverse transcription‐polymerase chain reaction (RT‐PCR) was performed on the Roche Light Cycler 2.0 detection system (Roche Diagnostic GmbH, Minato‐ku, Tokyo, Japan). Specific primers for the target genes were procured from Takara Bio Inc. (Otsu, Shiga, Japan). Gene expression levels were measured using the SYBR Green PCR Kit (Roche) following the manufacturer's protocol.

### 2.9 Apoptosis Assays

Apoptosis in MM cells treated with defined concentrations of selinexor and/or venetoclax was assessed using the Annexin V/Propidium Iodide (PI) binding assay (BD Biosciences) according to the manufacturer's guidelines. Cell suspensions were incubated with Annexin V and PI for 15 min at room temperature in the dark. Fluorescence was measured using flow cytometry (BD Biosciences), analyzing at least 10,000 cells per sample.

### 2.10 Mitochondrial Membrane Potential Assay

According to the manufacturer's instructions, the mitochondrial membrane potential (MMP) was evaluated using the JC‐1 MitoMP Detection Kit (Dojindo Laboratories). MM cells were exposed to selinexor and/or venetoclax for 48 h. Following incubation, JC‐1 monomers and aggregates were analyzed to detect changes in MMP using an EnSpire Multimode Plate Reader (PerkinElmer).

### 2.11 Statistical Analyses

Data analysis and visualization were conducted using Prism 10 software (GraphPad Software, San Diego, CA, USA). Statistical significance for pairwise comparisons was determined using a two‐tailed Student's t‐test. Results are expressed as means ± standard deviations (SDs) and were analyzed using one‐way analysis of variance (ANOVA). Tukey's or Dunnett's post hoc tests were used for multiple group comparisons, whereas a two‐tailed t‐test was used for comparisons between two groups. Significance levels were denoted as *p < 0.05, **p < 0.01, ***p < 0.001, and ****p < 0.0001.

## 3 Results

### 3.1 Expression Analysis of BCL2 Family Genes Under Hypoxic Conditions and Disease Progression

**FIGURE 1:**
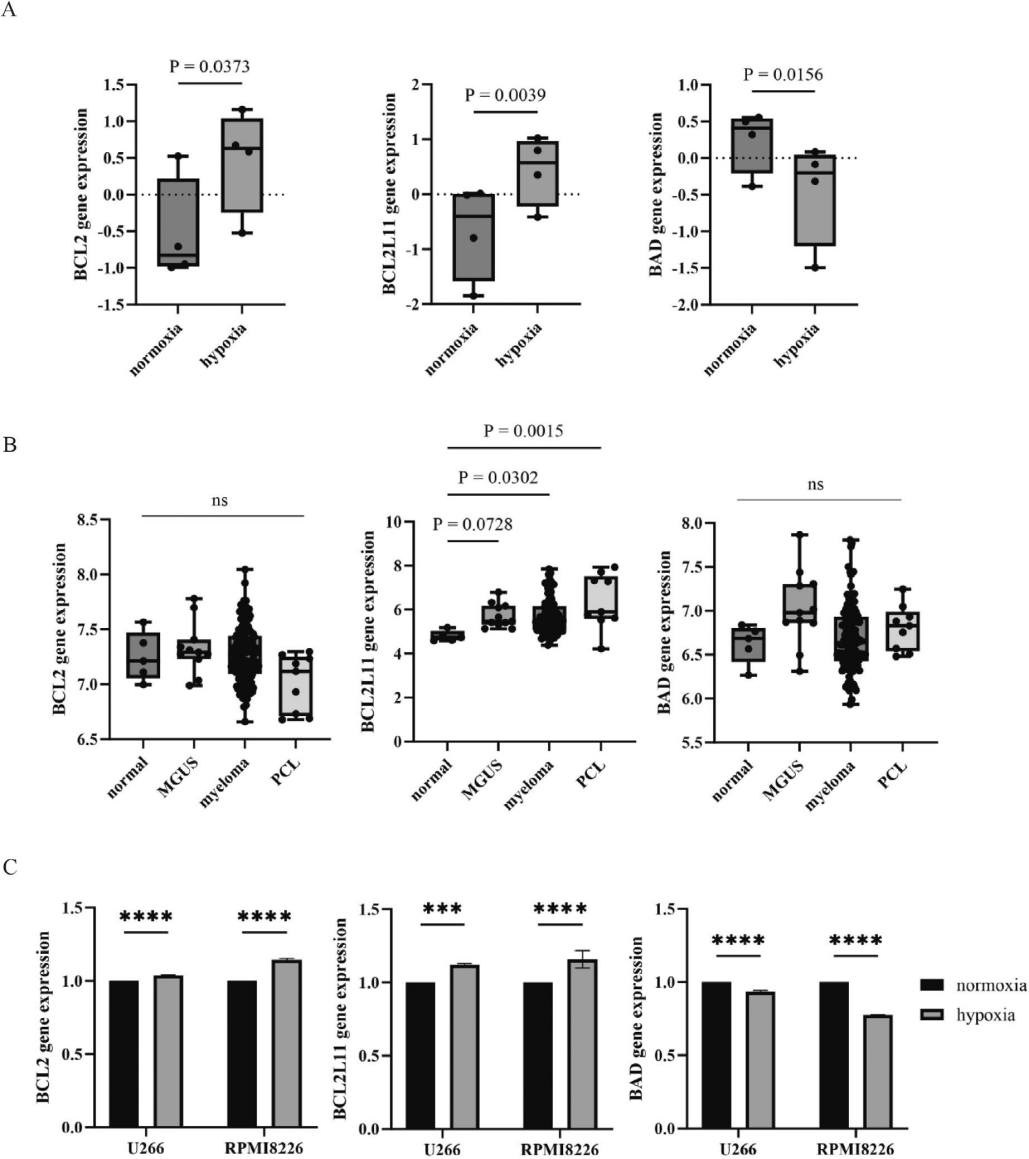
Expression of BCL2‐related genes during myeloma progression and under hypoxic conditions. (A) *BCL2, BCL2L11*, and *BAD* expression levels were validated using data from the GEO database, GSE80140. The results indicate statistical significance compared to normoxia. (B) *BCL2, BCL2L11*, and *BAD* expression levels were validated using data from the GEO database, GSE13591. Statistical significance compared to normal is indicated. (C) Total RNA was extracted from MM cell lines under normoxic and hypoxic conditions. *BCL2*, *BCL2L11*, and *BAD* gene expressions were analyzed using RT‐PCR.

We initially explored the expression levels of *BCL2*, *BCL2L11*, and *BAD* using publicly available data from the GEO database. Analysis of the dataset GSE80140 under hypoxic conditions revealed an upregulation of *BCL2* and *BCL2L11*, while *BAD* expression was downregulated (Figure 1A). Subsequently, we examined gene expression profiles in samples from patients with MGUS, MM, and PCL using data from the GSE13591 dataset. There were no significant changes in *BCL2* and *BAD* expression in these samples compared to normal controls (Figure 1B). However, *BCL2L11* expression was notably elevated across MGUS, MM, and PCL patient samples (Figure 1B). To further validate these findings, we assessed the expression of *BCL2*, *BCL2L11*, and *BAD* in MM cell lines under hypoxic conditions. Consistent with the GEO data, we observed increased expression of *BCL2* and *BCL2L11* under hypoxia, while *BAD* expression was reduced (Figure 1C).

### 3.2 Enhanced Activity of BCL2 Inhibitor, Venetoclax Under Hypoxia

**FIGURE 2:**
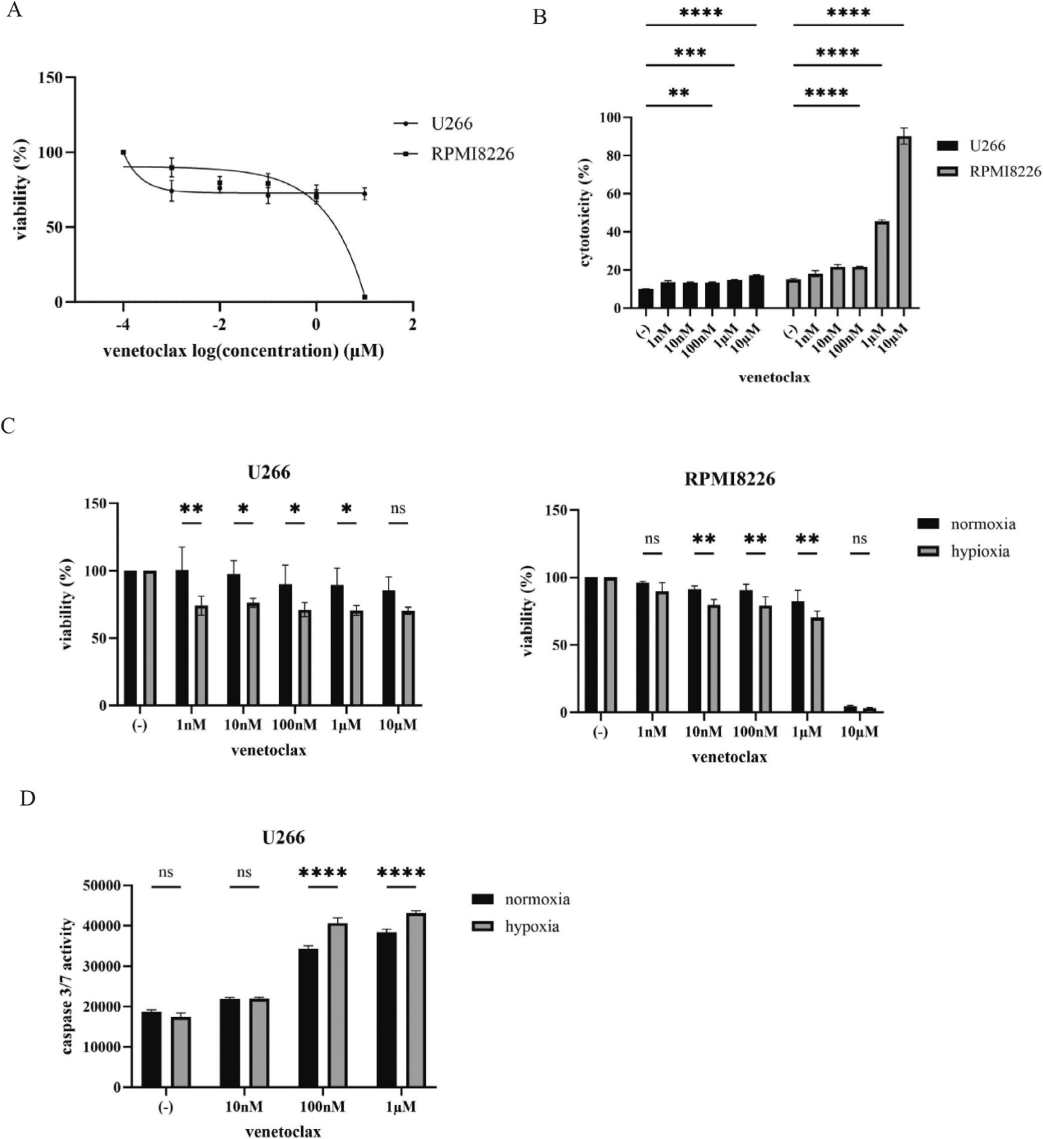
Effects of venetoclax on the MM cell lines. (A) MM cell lines were cultured in RPMI 1640 medium supplemented with 10% FBS, respectively, and with venetoclax for 72 h. Cell growth was evaluated with a cell counting kit‐8. (B) The MM cell lines were treated with venetoclax for 48 h. The cytotoxicity was analyzed using a Cytotoxicity LDH Assay kit. * Data were normalized to untreated controls and presented as mean ± standard deviation. **p < 0.01, ***p < 0.001, and ****p < 0.0001, compared with the control. (C) MM cell lines were cultured with venetoclax for 72 h under normoxic and hypoxic conditions. Cell growth was evaluated with a cell counting kit‐8. *p < 0.05,**p < 0.01, compared to normoxic conditions. (D) U266 cells were incubated with an indicated concentration of venetoclax for 48 h under normoxic and hypoxic conditions. Caspase 3/7 activity was evaluated. ****p < 0.0001, compared to normoxic conditions. ns: Not significant.

Venetoclax, a selective BCL2 inhibitor, was assessed for its efficacy in reducing cell viability [15]. Our results demonstrated that venetoclax decreased cell viability in a dose‐dependent manner, and its cytotoxic effects were similarly enhanced with increasing drug concentrations (Figure 2A,B). Given the observed upregulation of *BCL2* gene expression under hypoxic conditions, we further investigated the activity of venetoclax in a hypoxic environment. We found that venetoclax exhibited greater efficacy under hypoxia than normoxia, with a more pronounced reduction in cell viability (Figure 2C). Moreover, we observed a significant increase in caspase‐3/7 activity in hypoxic conditions, suggesting that the enhanced sensitivity to venetoclax under hypoxia is mediated by activating apoptotic pathways (Figure 2D). These findings indicate that hypoxia potentiates venetoclax activity, likely due to the upregulation of BCL2.

### 3.3 Activity of Selinexor in Multiple Myeloma Cell Lines

**FIGURE 3:**
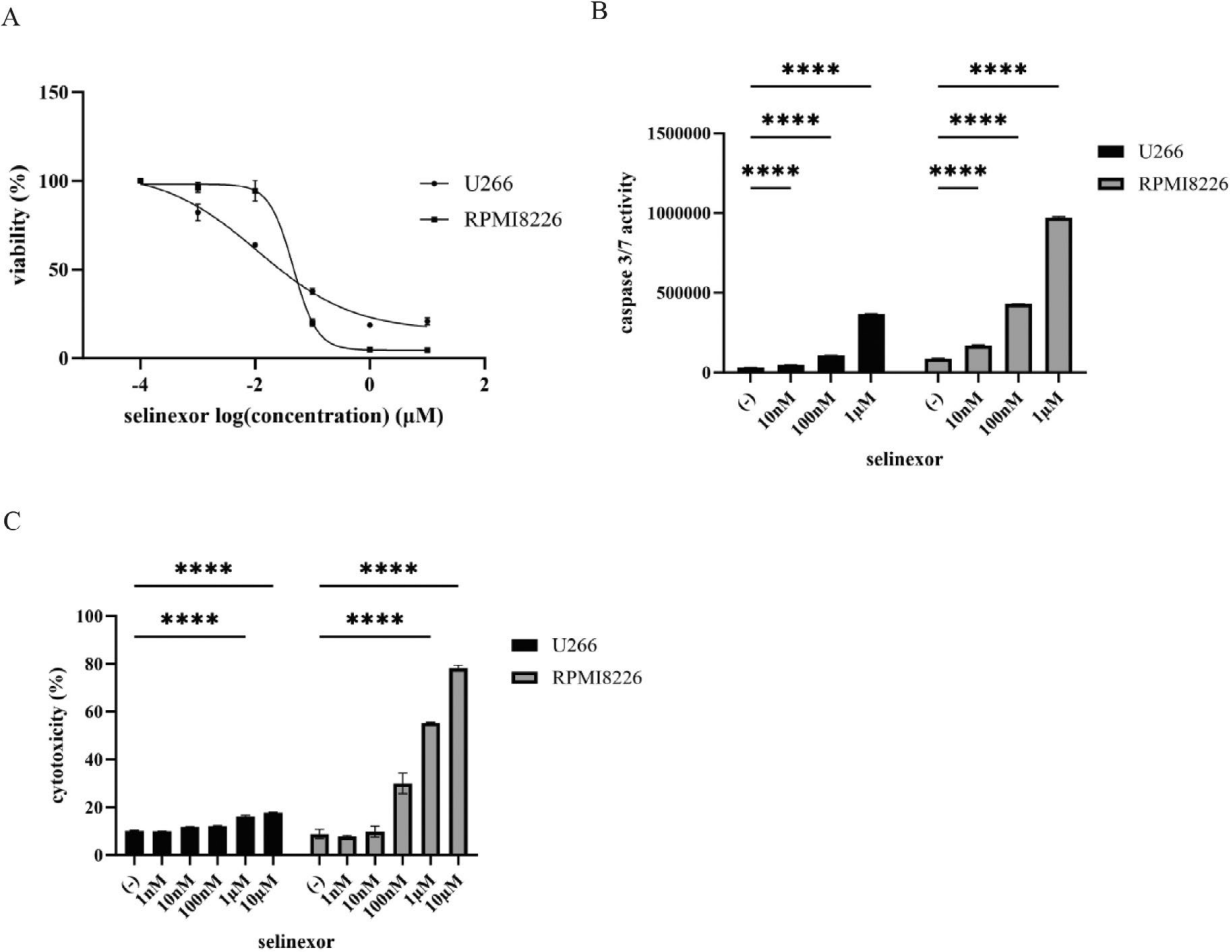
Effects of selinexor on the MM cell lines. (A) MM cell lines (U266 and RPMI8226) were cultured in RPMI 1640 medium with the indicated concentration of selinexor for 72 h. Cell viability was evaluated using a cell counting kit‐8. (B) MM cell lines (U266 and RPMI8226) were treated with the indicated concentration of selinexor for 48 h. Caspase 3/7 activity was evaluated. ****p < 0.0001 vs. the control. (C) MM cell lines (U266 and RPMI8226) were treated with the specified concentrations of selinexor for 48 h. The cytotoxicity was subsequently assessed utilizing a Cytotoxicity LDH Assay kit. ****p < 0.0001, compared to control.

Selinexor, an XPO1 inhibitor, is clinically used to treat patients with refractory MM.^7^ To investigate its therapeutic potential, we evaluated the activity of selinexor in MM cell lines. Our results demonstrated a dose‐dependent reduction in cell viability following treatment with selinexor (Figure 3A). Additionally, selinexor treatment led to a significant increase in caspase‐3/7 activity (Figure 3B). Cytotoxicity was also elevated in response to increasing drug concentrations (Figure 3C). These findings suggest that selinexor exerts potent anti‐myeloma effects by promoting apoptotic cell death.

### 3.4 Anti‐Myeloma Effects of Selinexor With Venetoclax Under Hypoxia

**FIGURE 4:**
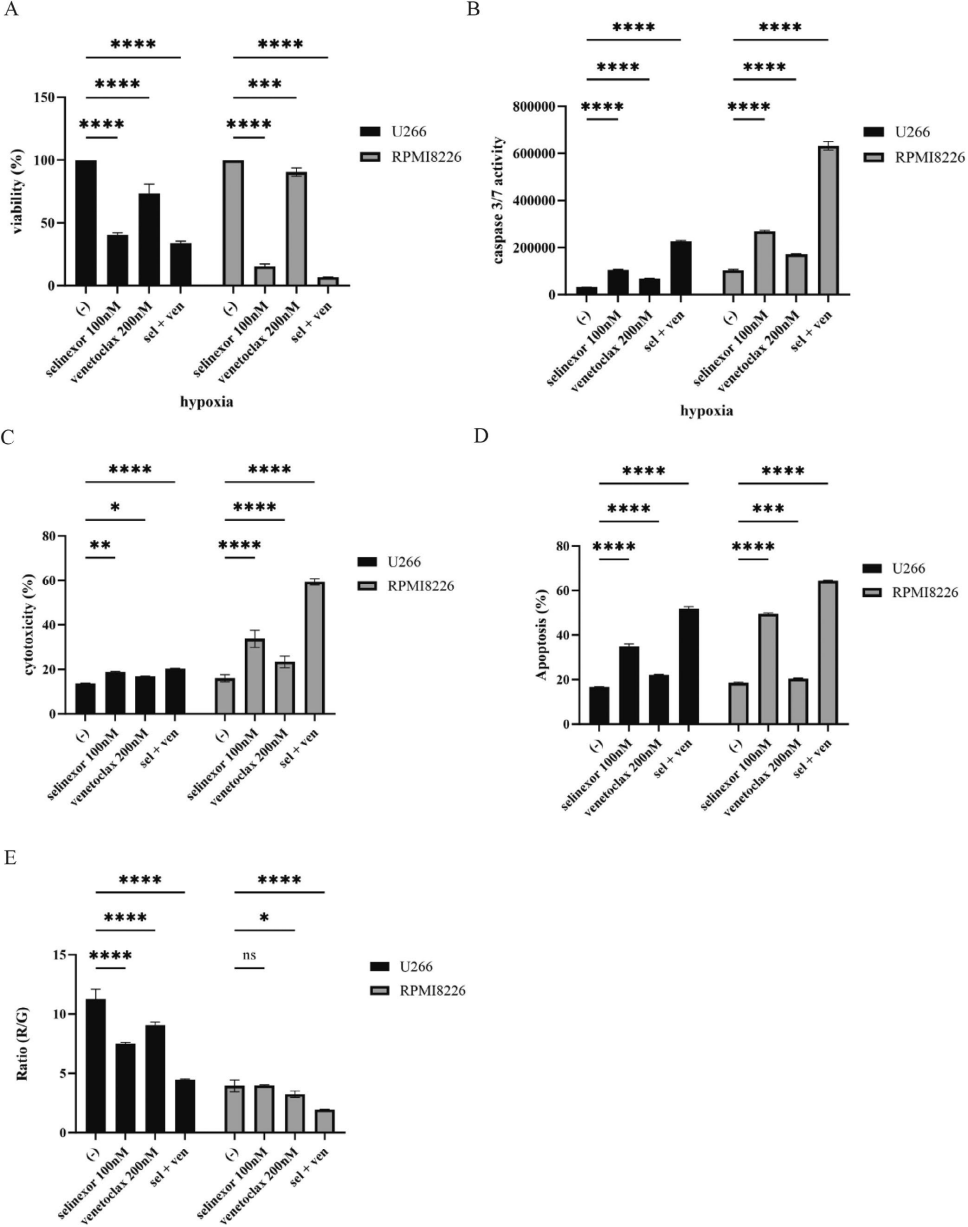
The activity of selinexor and venetoclax on the MM cell lines. (A‐D) U266 and RPMI 8226 cells were cultured with selinexor and/or venetoclax for 48 or 72 h. (A) Cell viability, (B) caspase 3/7 activity, (C) cytotoxicity, and (D) apoptosis were evaluated. Significance was expressed as *p < 0.05, **p < 0.01, ***p < 0.001, ****p < 0.0001, and ns: Not significant vs. the control. (E) U266 and RPMI 8226 cells were treated with selinexor and/or venetoclax for 48 h. The MMP was analyzed using a JC‐1 MitoMP Detection Kit. Significance is expressed as *p < 0.05, **p < 0.01, and ns: Not significant vs. the control.

Next, we investigated the combined effects of selinexor and venetoclax on MM cell lines under hypoxic conditions. Combining selinexor and venetoclax resulted in a more significant reduction in cell viability than treatment with either drug alone (Figure 4A). We observed that the combination treatment significantly increased caspase‐3/7 activity, cytotoxicity, and the percentage of apoptotic cells (Figure 4B,C 4D). Further analysis revealed that the combination of selinexor and venetoclax also disrupted the MMP, indicating mitochondrial dysfunction as a potential mechanism underlying the enhanced apoptotic response (Figure 4E). These results suggest that the combination of selinexor and venetoclax exerts anti‐myeloma effects under hypoxic conditions by promoting apoptosis.

### 3.5 Efficacy of Selinexor and Venetoclax Combination in Overcoming Bortezomib Resistance and Primary Plasma Cell Leukemia Cells

**FIGURE 5:**
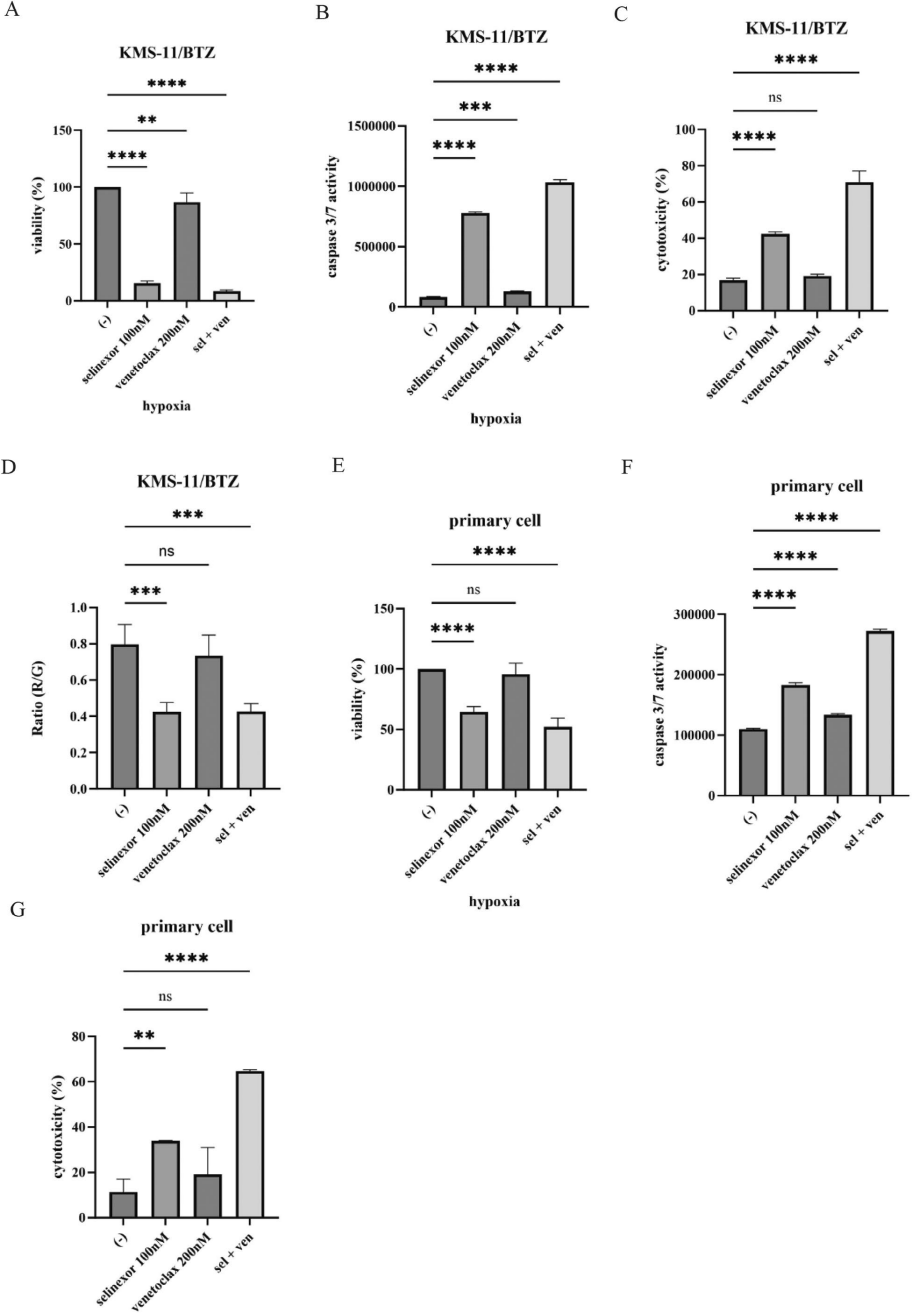
Efficacy of selinexor and venetoclax on the bortezomib‐resistant MM cell line and primary PCL sample. (A‐C) KMS‐11/BTZ cells were cultured with selinexor and/or venetoclax for 48 or 72 h. (A) Cell viability, (B) caspase 3/7 activity, (C) cytotoxicity, and (D) apoptosis were evaluated. **p < 0.01, ***p < 0.001, ****p < 0.0001, and ns: Not significant vs. the control. (D) KMS‐11/BTZ cells were treated with selinexor and/or venetoclax for 48 h. The MMP was analyzed using a JC‐1 MitoMP Detection Kit. Significance is expressed as ***p < 0.001, and ns: Not significant vs. the control. (E–G) Primary PCL cells were cultured with selinexor and/or venetoclax for 48 or 72 h. (E) Cell viability, (F) caspase 3/7 activity, and (G) cytotoxicity were evaluated. **p < 0.01, ****p < 0.0001, and ns: Not significant vs. the control.

To investigate the therapeutic potential of selinexor and venetoclax, we examined their effects on the bortezomib‐resistant MM cell line, KMS‐11/BTZ. Combining selinexor and venetoclax effectively reduced cell viability in these resistant cells (Figure 5A). We observed that caspase‐3/7 activity was significantly elevated, and cytotoxicity was markedly enhanced (Figure 5B,C). Moreover, the MMP was reduced, suggesting mitochondrial dysfunction as a contributing factor to the induced cell death (Figure 5D). We extended these findings by evaluating the combination treatment in primary PCL samples. A primary PCL cell with a complex karyotype (41–43 chromosomes) was also prepared for further study. The karyotypic abnormalities identified included add(2)(p14), add(3)(p24), add(7)(p21), −8, add(10)(p14), −13, −15, and +1–2 marker chromosomes (mar). These structural and numerical abnormalities are indicative of a high‐risk cytogenetic profile in PCL. Consistent with the results in MM cell lines, the combination of selinexor and venetoclax reduced cell viability in PCL samples (Figure 5E). Furthermore, there was a significant increase in caspase‐3/7 activity and cytotoxicity (Figure 5F,G). These findings suggest that the combination of selinexor and venetoclax is effective in bortezomib‐resistant MM cells and primary PCL samples.

## 4 Discussion

This study demonstrated that the BCL2 inhibitor, venetoclax, is effective against myeloma cells under hypoxic conditions. Considering the naturally hypoxic nature of the BM microenvironment, this discovery holds critical therapeutic implications.^10^ Data from the GEO database revealed that the expression of BCL2 is elevated in myeloma cells exposed to hypoxia compared to normoxia, suggesting a potential mechanism for increased survival of these cells in such conditions. Furthermore, co‐treatment with venetoclax and the XPO1 inhibitor, selinexor, showed enhanced efficacy in myeloma cells under hypoxic conditions, indicating that this combination therapy may be a promising approach for targeting myeloma cells in the hypoxic BM niche.

Our analysis further revealed differential gene expression patterns in response to hypoxia in myeloma cells. BCL2 is part of a family of regulatory proteins that promote or inhibit cell death [16]. The balance between pro‐apoptotic and anti‐apoptotic proteins influences cell fate, which differs among various cell types [17]. Interestingly, the expression of the pro‐apoptotic *BCL2L11* gene (BIM) was significantly elevated under hypoxic conditions, while the expression of the *BAD* gene was reduced. *BCL2* and *BAD* gene expression did not differ between normal and MM cells, suggesting these genes may not contribute to the malignancy‐specific adaptation under hypoxia. In contrast, BCL2L11 expression was higher in MM cells compared to normal cells. This indicates that BCL2L11 may also play a critical role in the response of MM cells to the hypoxic BM microenvironment, potentially contributing to their survival and resistance mechanisms.

Co‐treatment with selinexor and venetoclax induced significant cytotoxicity in myeloma cells. This combination therapy led to a marked reduction in MMP, indicating mitochondrial dysfunction as a key mechanism of cell death. Moreover, the proportion of apoptotic cells was substantially increased following treatment, confirming the pro‐apoptotic effects of selinexor and venetoclax in this context. These findings suggest that the dual inhibition of XPO1 and BCL2 effectively enhances apoptosis and disrupts mitochondrial integrity in myeloma cells, making this combination a promising therapeutic strategy for targeting myeloma.

The combination of selinexor and venetoclax was effective against bortezomib‐resistant myeloma cell lines, demonstrating its potential to overcome resistance to standard therapies. Furthermore, this co‐treatment showed significant efficacy in primary PCL cells under hypoxic conditions. These results highlight the therapeutic promise of targeting the XPO1 and BCL2 pathways in drug‐resistant myeloma and more aggressive forms like PCL, particularly within the hypoxic BM microenvironment.

Venetoclax is a potent, highly selective oral BCL2 inhibitor that triggers apoptosis in MM cells [15]. A phase 1 trial demonstrated that combining venetoclax with bortezomib and dexamethasone exhibited promising clinical efficacy and acceptable safety and tolerability [18]. The t(11;14) translocation defines a distinct MM subgroup with BCL2 dependency [12]. However, there was an increased mortality signal in the randomized BELLINI trial that was primarily driven by non‐t(11;14) patients [19]. Based on current evidence, venetoclax is being considered in clinical practice for the treatment of relapsed/refractory t(11;14) myeloma. It enables effective targeted therapy using BCL2 inhibitors like venetoclax, particularly in relapsed/refractory cases, highlighting its importance in risk stratification and personalized treatment strategies. Thus, combining selinexor and venetoclax could be a promising therapeutic approach for MM patients t(11;14) by targeting BCL2 dependency and nuclear export pathways to improve treatment outcomes [20]. Clinical trials have highlighted the advantages of adding venetoclax to a carfilzomib and dexamethasone regimen, encouraging further evaluation of venetoclax at daily doses of 400–800 mg in combination therapy for patients with t(11;14)‐positive relapsed/refractory MM [21]. Badawi M et al. support the further exploration of venetoclax at an 800 mg once‐daily dose in combination with dexamethasone for t(11;14)‐positive patients, showing improved efficacy without a significant rise in safety‐related adverse events [22].

The combination of BCL2 and XPO1 inhibitors shows promise but may be associated with significant toxicities. Venetoclax can cause tumor lysis syndrome, neutropenia, and gastrointestinal issues; Selinexor often leads to fatigue, cytopenias, and hyponatremia [23, 24]. Their combination may exacerbate hematologic toxicities, increasing infection and bleeding risks. Close monitoring and supportive care are essential to manage these effects.

The cell lines utilized in this study do not possess the t(11;14) translocation, a recognized biomarker for venetoclax responsiveness. Nonetheless, emerging evidence indicates that BCL2 dependency in MM is not solely associated with t(11;14) but may also be influenced by other molecular factors, such as the relationship between anti‐ and pro‐apoptotic BCL2 family members under hypoxia [25, 26]. Consequently, this study offers valuable insights into the potential broader applicability of venetoclax, particularly under hypoxic conditions where apoptotic regulation is altered.

BCL2 expression is elevated in hypoxia compared to normoxia due to molecular and cellular adaptive mechanisms that support cancer cell survival. One key factor is HIF1α, which is stabilized under hypoxic conditions and regulates genes involved in apoptosis resistance. HIF1α regulates BCL2‐related genes, leading to their upregulation to promote cell survival [27]. Consistent with this, our study observed increased BCL2 expression under hypoxic conditions compared to normoxia.

Our findings highlight the therapeutic potential of combining selinexor and venetoclax for MM and PCL. The combination effectively enhances apoptotic responses, disrupts mitochondrial function, and overcomes bortezomib resistance, particularly under hypoxic conditions. These results underscore the synergistic anti‐MM effects of this combination, offering a promising strategy to target hypoxia‐driven resistance and improve outcomes in refractory or high‐risk plasma cell malignancies, particularly in cases with the t(11;14) translocation. Further clinical validation is still warranted.

## Author Contributions

**Seiichi Okabe, Yuya Arai, and Yuko Tanaka:** conceptualization. **Seiichi Okabe and Yuya Arai:** methodology. **Seiichi Okabe:** investigation, visualization. **Seiichi Okabe and Akihiko Gotoh:** writing – original draft.

## Ethics Statement

Ethical approval for the study was granted by the Ethics Committee of Tokyo Medical University (approval number: T2023‐0105).

## Conflicts of Interest

Akihiko Gotoh received research funding from Ono Pharmaceutical Co. Ltd.; Taiho Pharmaceutical Co. Ltd.; Chugai Pharmaceutical Co. Ltd.; Otsuka Pharmaceutical Co. Ltd.; and Asahi Kasei Co. Ltd. Akihiko Gotoh received honoraria from Novartis Pharma K.K.; Alexion Pharmaceuticals Inc., Eisai Co. Ltd.; Ono Pharmaceutical Co. Ltd.; Taiho Pharmaceutical Co. Ltd.; Takeda Pharmaceutical Co. Ltd.; Nippon Shinyaku Co. Ltd.; Chugai Pharmaceutical Co. Ltd.; Otsuka Pharmaceutical Co. Ltd.; Sumitomo Pharma Co. Ltd.; Daiichi Sankyo Co. Ltd.; Nihon Pharmaceutical Co. Ltd.; Kyowa Kirin Co. Ltd.; Janssen Pharmaceutical K.K.; Pfizer Japan Inc.; Sanofi K.K. and Asahi Kasei. Akihiko Gotoh received consulting fees from PharmaEssentia Japan K.K.; Chugai Pharmaceutical Co. Ltd.; Alexion Pharmaceuticals Inc.; and Asahi Kasei. In addition, Akihiko Gotoh participated in the data safety monitoring board or advisory board of PharmaEssentia Japan K.K.; Chugai Pharmaceutical Co. Ltd.; and Alexion Pharmaceuticals Inc.

## Data Availability

The data that support the findings of this study are available from the corresponding author upon reasonable request.
